# Diagnostic Value of microRNA for Alzheimer’s Disease: A Systematic Review and Meta-Analysis

**DOI:** 10.3389/fnagi.2016.00013

**Published:** 2016-02-09

**Authors:** Yong-Bo Hu, Chun-Bo Li, Ning Song, Yang Zou, Sheng-Di Chen, Ru-Jing Ren, Gang Wang

**Affiliations:** ^1^Department of Neurology, Neuroscience Institute, Ruijin Hospital, Shanghai Jiao Tong University School of Medicine, Shanghai, China; ^2^Shanghai Key Laboratory of Psychotic Disorders, Shanghai Mental Health Center, Shanghai Jiao Tong University School of Medicine, Shanghai, China; ^3^St George Hospital, Sydney, NSW, Australia

**Keywords:** Alzheimer’s disease, miRNA, diagnostic value, systematic review, meta-analysis

## Abstract

Sound evidence indicates that microRNAs (miRNAs) are aberrantly expressed in Alzheimer’s disease (AD) patients. We performed a systematic review and meta-analysis to investigate the role of miRNA in AD pathogenesis and their clinical diagnostic value; a systematic review of literature and meta-analysis of clinical trials were performed. In the systematic review, 236 papers were included, and we reviewed the dysregulated miRNA expression in different parts of AD patients in order to identify the relationship between aberrantly expressed miRNAs and AD pathology. In the subsequent meta-analysis, seven studies were statistically analyzed with the following results: pooled sensitivity 0.86 (95%CI 0.79–0.90), pooled specificity 0.87 (95%CI 0.72–0.95), diagnostic odds ratio (28.29), and the area under the curve (0.87). In conclusion, our review indicated that aberrant expression of various miRNAs plays an important role in the pathological process of AD, and statistical analysis of quantitative studies reveal the potential value of specific miRNAs in the diagnosis of AD.

## Introduction

Alzheimer’s disease (AD) is the most common form of dementia among the aging population, characterized pathologically by the progressive accumulation of extracellular amyloid-beta (Aβ) plaques and intracellular neurofibrillary tangles (NFT) (Goedert and Spillantini, 2006). The insidious nature of its onset may delay the diagnosis of AD for several years, and late diagnosis leads to poor patient prognosis. Currently, diagnostic approaches to AD involve clinical findings, neuropsychological testing, and neuroimaging assessment, but there lacks a method that has high sensitivity and specificity, especially for early diagnosis (Huang and Mucke, 2012). Even though many studies suggest that Aβ imaging using positron emission tomography (PET) scanning and Aβ levels measurement in CSF and serum may serve as promising methods in diagnosis, the high cost and invasive nature preclude their utility for routine clinical tests.

Recently, increasing evidence indicates that microRNA (miRNA) plays a major role in the brain, involving in neural development and differentiation (Jiang et al., 2013; Lin et al., 2014). microRNAs (miRNAs) are conserved small non-coding RNAs and modulate gene expression negatively at the post-transcriptional level. Approximately 70% of the currently identified ­miRNAs are expressed in the brain (Lausted et al., 2014; Bennett et al., 2015). Therefore, miRNAs may serve a potential role in monitoring neurodegenerative processes, and specific miRNAs may correlate with certain neurodegenerative diseases. They may act as biomarkers to discern and identify the neurodegenerative process in disorders like AD before clinical symptoms and functional decline become apparent.

We conducted a systematic review of related qualitative and quantitative studies to collaborate the findings on changes of miRNA expression in different parts of the brains in patients with AD. We also evaluated the diagnostic value of miRNAs for AD through completing this meta-analysis.

## Material and Methods

### Literature Search

We searched the following electronic bibliographic databases: MEDLINE, EMBASE, The Cochrane Library (Cochrane Database of Systematic Reviews), Cochrane Central Register of Controlled Trials (CENTRAL), Health Technology Assessment Database, and Web of Science (science and social science citation index). Furthermore, we searched the Chinese databases of China National Knowledge Infrastructure (CNKI), Wan Fang Data, Technology of Chongqing VIP database, and SinoMed for related studies. The search strategy included terms relating to or describing the clinical trials of diagnosis. The search terms are adapted for use with other bibliographic databases in combination with database-specific filters for controlled trials, where these are available. The language was restricted to English and Chinese and the publication date from January 1990 to June 2015. The search terms were “Alzheimer’s disease/AD/dementia of the Alzheimer’s type/Alzheimer dementia,” “microRNA/miRNA,” “diagnosis,” “sensitivity,” and “specificity.”

The protocol has been registered in PROSPERO, an international prospective register of systematic review with a registration No. CRD42015015925.

### Inclusion and Exclusion Criteria

In the systematic review, we included all quantitative and qualitative studies, reports, letters, reviews, editorial articles, or conference abstracts examining the relationship between miRNA expression and AD. Those literatures were included regardless of quality.

For meta-analysis, a study was included if it met all of the following criteria: (1) randomized trials or observational studies (including cohort and case-control studies); (2) all included patients were diagnosed with AD using clinically recognized diagnostic criteria; (3) clinical studies on evaluation of miRNAs in the diagnosis of AD; (4) inclusion of sufficient data on the size of samples and the sensitivity and specificity data. Publications with limited data information or overlap of patient selection with other included studies were excluded.

### Data Extraction and Quality Assessment of Included Studies

In meta-analysis, the final included studies were independently assessed by two reviewers using a standardized form. Information retrieved from these articles included: the first author, publication year, sample size, specimen, miRNA expression signature, sensitivity, and specificity data. The methodological quality of included studies were assessed by QUADAS (quality assessment for studies of diagnostic accuracy), which is an evidence-based quality assessment tool for systematic reviews of diagnostic accuracy studies (Whiting et al., 2006, 2011; Mann et al., 2009).

### Statistical Analysis of Meta-Analysis

The true positives (TP, the individual has the condition and tests positive for the condition), false positives (FP, the individual does not have the condition but tests positive for the condition), true negatives (TN, the individual does not have the condition and tests negative for the condition), and false negatives (FN, the individual has the condition but tests negative for the condition) were calculated with obtained data after constructing 2 × 2 contingency tables. The following indices were computed: pooled sensitivity, pooled specificity, positive likelihood ratio (PLR), negative likelihood ratio (NLR), diagnostic odds ratio (DOR), and their corresponding 95% confidence intervals (CI). Fagan’s nomogram was used for post-test probability calculations. Cochran-*Q* value and *I*^2^ test were used to assess heterogeneity across studies. Deek’s funnel plots were adopted to investigate publication bias. All analyses were performed with the following software programs: Stata, review manager 5.3 and Meta-Disc 1.4. All statistical tests were two sided, and the significance level was set at *p* < 0.05.

## Results

### Systematic Review: Identified Relationship between miRNA and AD

#### miRNA Dysregulation Found in Different Human Tissues/Samples (Table 1)

**Table 1:** **Systematic review: miRNA dysregulation in different parts**.

Brain-based miRNA		CSF-based miRNA	Blood-based miRNA		
Cortex	Hippocampus		Plasma	Serum	PBMC
miR-129-5p	miR-132-3p	miR-34a, miR-125b	miR-34a/c	miR-137	miR-34a
miR-27a-3p	miR-128	miR-146a, miR-29a	miR-146a	miR-18c	miR-181b
miR-92b-3p	miR-136-5p	miR-27a-3p	miR-128	miR-9	miR-200a
miR-200a	miR-138-5p	miR-24, miR-126	miR-132	miR-29a	let-7f
miR-148	miR-145	miR-10a/b, miR-16	miR-29a/b	let-7f	
miR-370	miR-124-3p	miR-138, miR-141	miR-874	miR-29b	
miR-409-5p	miR-129-5p	miR-143, miR-151	miR-134	miR-126	
miR-127-5p	miR-129-2-3p	miR-181a/c	miR-323-3p	miR-34a	
miR-496	miR-487	miR-191, miR-194	miR-382	miR-181b	
miR-633	miR-370	miR-195, miR-204	miR-137		
miR-874	miR-409-5p	miR-205, miR-214	miR-181c		
	miR-487	miR-221, miR-338			
Lau et al. (2013), Delay et al. (2012), Bekris et al. (2013)	Lau et al. (2013), Delay et al. (2012)	Bekris et al. (2013), Cogswell et al. (2008), Kiko et al. (2014), Muller et al. (2014), Sala Frigerio et al. (2013), Burgos et al. (2014)	Kumar et al. (2013), Bekris et al. (2013), Bhatnagar et al. (2014), Kiko et al. (2014)	Leidinger et al. (2013), Cheng et al. (2014), Tan et al. (2014b), Tan et al. (2014a), Geekiyanage et al. (2012)	Schipper et al. (2007)

#### Peripheral Blood

Peripheral blood sampling is a minimally invasive procedure that is simple and inexpensive to perform. The remarkable stability despite the abundance of endogenous RNAs in biofluid allows postulation of the pathogenesis in AD, as well as opening up an avenue for developing new diagnostic tests in clinical practice (Kiko et al., 2014). In 2007, Schipper and colleagues reported that expression of nine miRNAs was increased in the peripheral blood mononuclear cells of AD patients, and subsequently, Geekiyanage et al. (2012) discovered that miR-137, miR-181c, miR-9, and miR-29 were downregulated in the serum of AD patients as well as AD mouse models. In patients with mild cognitive impairment (MCI), there appears to be an increased level of brain enriched miRNAs from the miR-132 and miR-134 families compared to age matched controls (Sheinerman and Umansky, 2013). Moreover, Tan et al. (2014b) applied high through sequencing to genome-wide serum miRNAs and identified miR-342-3p as a potential serum biomarker in aiding the diagnosis of AD, with a sensitivity of 81.5% and specificity of 70.1%. These explorations suggested a promising role for serum miRNAs to serve as a diagnostic marker for AD, although further analysis and validation of these results would be required.

#### Cerebrospinal Fluid

Apart from blood-based analysis, miRNA from cerebrospinal fluid (CSF) has gained increasing attention in studies exploring for potential AD biomarkers, as Aβ, total tau, and phosphorylated tau levels in CSF can support the clinical diagnosis of AD with acceptable sensitivity and specificity (Ghidoni et al., 2011; Blennow and Zetterberg, 2013; Lleo et al., 2015). Cogswell et al. (2008) examined post-mortem CSF and found 60 miRNAs expression levels were significantly different between AD patients and normal controls. However, in ante-mortem cell free CSF, most selected miRNAs were undetectable or only minimally detectable, producing a highly variable result when compared to former reports (Muller et al., 2014). Nevertheless, in the same study, miR-146a was significantly decreased in CSF of AD patients and low level of 146a was associated with disease progression (Muller et al., 2014).

#### Brain Tissues

Five hippocampal miRNAs (miR-370, miR-328, miR-15a, miR-138, and miR-132) were identified with sound sensitivity and specificity in predicting AD plaques scores or Braak stages, suggesting that expression levels of dysregulated miRNAs may contribute to the AD neuronal pathology (Absalon et al., 2013; Bekris et al., 2013; Lau et al., 2013). The trend of miRNAs expression across the Braak stages is shown in Figure S1 in Supplementary Material. Moreover, variations in miRNAs expression in different brain regions may represent specific patterns of pathology (Hebert et al., 2010), which may be useful in characterizing specific miRNA biomarkers in AD diagnosis. Most existing studies examined miRNA in the hippocampus or cerebral cortex in the post-mortem setting and obtaining live tissue through biopsy would prove to be challenging.

### miRNA Dysregulation Involved in AD Pathogenic Protein or Process

Traditionally, we focused on the proteins changes related directly to pathogenesis for the onset and development of AD. Recently, however, a large-scale annotation of human genome provided evidence that miRNAs play an important role in the molecular pathogenesis of AD, both genetic and sporadic. miRNAs serve as important gene regulators and are critical for gene expression and demonstrated potential for novel therapeutic avenues in AD treatment (Berezin et al., 2014)

The single-stranded activated miRNAs can target complementary regions of message transcripts through base pairing mechanism, which results in either translational inhibition or degradation of mRNA (Wang, 2010). To date, several studies have demonstrated an altered expression of miRNAs in AD patients and how these alterations may play a direct role in moderating the expression of AD-related genes and subsequent phenotypic manifestation (Kiko et al., 2014). Even though a single miRNA can target hundreds of genes, the genes co-regulated by a set of miRNAs generally encode for a related network of functionally associated molecules. Hence, even small changes in the expression level of miRNAs could affect a wide range of signaling pathways involved in cellular physiological functions.

A large number of exploratory studies attempted to identify biologically and physiologically relevant molecular networks in AD by searching for meaningful changes in miRNA expression and potential surrogate diagnostic biomarkers in AD. AD-related proteins such as amyloid precursor protein (APP), beta-secretase 1 (BACE1), presenilin-1 (PSEN1), and MAPT (tau) and neuronal apoptosis have been thoroughly investigated during this search (Table 2).

**Table 2:** **Systematic review: miRNA dysregulation linked to AD pathogenesis**.

AD pathology	miRNA	Reference
APP	miR-101, miR-17-5p, miR-106b, miR-107, miR-20a, miR-30, miR-143, miR-153, miR-193, miR-9, miR-16	Satoh (2012), Schonrock et al. (2010), Delay et al. (2011), Wang et al. (2008), Liu et al. (2012), Hebert et al. (2009), Chang et al. (2014)
BACE1	miR-29a/b, miR-9, miR-5a, miR-19b, miR-298, miR-285-5p, miR-486, miR-107, miR-195	Hebert et al. (2008), Long et al. (2014), Schonrock et al. (2010), Satoh (2012)
Tau	miR-132,miR-125b, miR-26b,miR-128, miR-922, miR-34, miR-15, miR-512	Absalon et al. (2013), Dickson et al. (2013), Banzhaf-Strathmann et al. (2014), Zhao et al. (2014), Dickson et al. (2013), Mezache et al. (2015)
PSEN1	miR-214, miR-153, miR-516a, miR-511, miR-128, miR-340, miR-335	Delay et al. (2012), Satoh (2012), Mallick and Ghosh (2011), Kalani et al. (2013)
Apoptosis	let-7, miR-15a, miR-29, miR-17-5p, miR-26b, miR-21, miR-191, miR-590-3p, miR-132, miR-212	Absalon et al. (2013), Tan et al. (2013), Wong et al. (2013), Villa et al. (2011)

### miRNA Differently Expressed According to Gender and ApoE

It is believed that AD may be more prevalent in females than males (van Harten et al., 2015). Given that many reports of miRNA dysregulation in AD patients, several groups of researchers’ studies have started exploring how gender affects miRNA expression. For example, in mice models of AD, Geekiyanage et al. (2012) found the expression of miR-137, miR-181c, and miR-29 were decreased in the cortex of female mice compared to the males. Subsequently, they proved that these three miRNAs were also downregulated in serum of probable AD and amnestic MCI mice. APOE allele is also considered to be an important risk factor for AD, as the prevalence of APOE ϵ4 is significantly higher in the AD group. In 2014, Cheng et al. identified four clusters of miRNA, which showed clear associations with APOE ϵ4 allele status. They further selected 16 miRNAs markers based on the contribution toward clinical classification of AD through random forest analysis. Furthermore, a model derived from the study incorporating selected miRNAs, age, gender, and APOE ϵ4 allele status had correctly diagnosed 13 out of 15 AD participants AD patients with high accuracy (87% sensitivity and 77% specificity)

## Meta-Analysis: Diagnostic Value of miRNA for AD

### Data Characteristics and Quality Assessment

**Figure 1:**
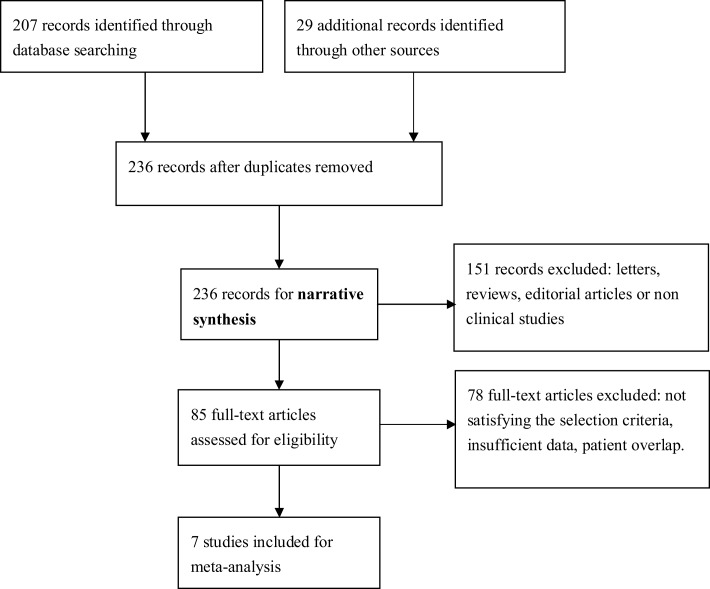
**Process of study selection**.

We identified seven English publications focusing on miRNAs expression for diagnosis of AD. The details of the selection process were presented in Figure 1.

A total of 418 cases and 442 controls were involved for analysis across the seven studies and the data characteristics with the QUADAS score of each study were listed in Table 3. All patients with AD were diagnosed with clinically recognized diagnostic criteria.

**Table 3:** **Summary of included studies**.

Study	Reference	No. of patients	No. of controls	Specimen	TP	FP	FN	TN	QUADAS	miRNA profile
1	Leidinger et al. (2013)	48	22	Plasma	44	1	4	21	10	miR-112, 161, 5010-3p, 26a-5p, 1285-5p, 151-3p
2	Tan et al. (2014a)	158	155	Serum	127	49	31	106	13	miR-98-5p, 885-5p, 483-3p, 191-5p, let-7d-5p
3	Cheng et al. (2014)	15	35	Serum	13	8	2	27	10	miR-1306-5p, 342-3p, 15b-3p
4	Tan et al. ([Bibr B47][Bibr B46])	105	150	Serum	85	48	20	102	12	miRNA-125b
5	Lau et al. (2013)	41	23	Hippocampus	37	0	4	23	13	miR-132-3p, 128, 136-5p, 138-5p, 124-3p, 129-5p
6	Muller et al. (2014)	20	30	CSF	12	2	8	18	13	miR-16
7	Kumar et al. (2013)	31	37	Plasma	29	2	2	35	11	miR-545-3p, let-7g-5p
Total		418	442							

The specimens used for miRNA analysis included CSF, hippocampus, plasma, and serum. The miRNAs expressions were detected by quantitative reverse transcription polymerase chain reaction (qRT-PCR) and *in situ* hybridization (ISH). After analyzing each study, their QUADAS scores ranged from 10 to 13.

### Heterogeneity Analysis and Diagnostic Value of miRNAs

**Figure 2:**
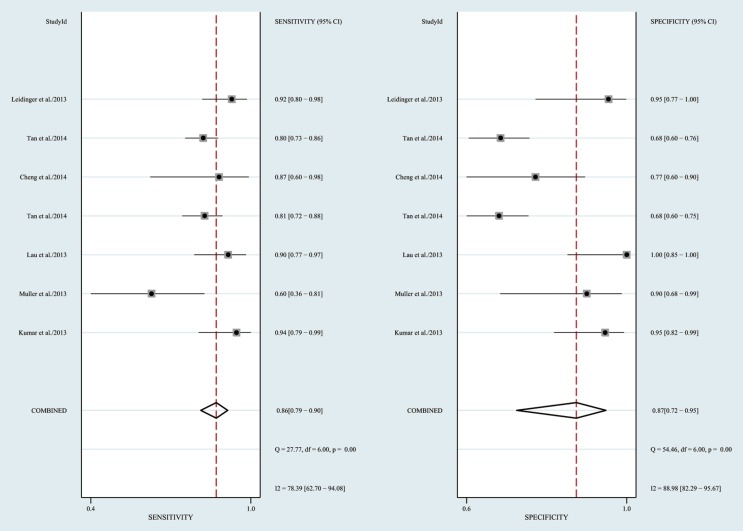
**Forrest plot of estimates of sensitivity and specificity**.

**Figure 3:**
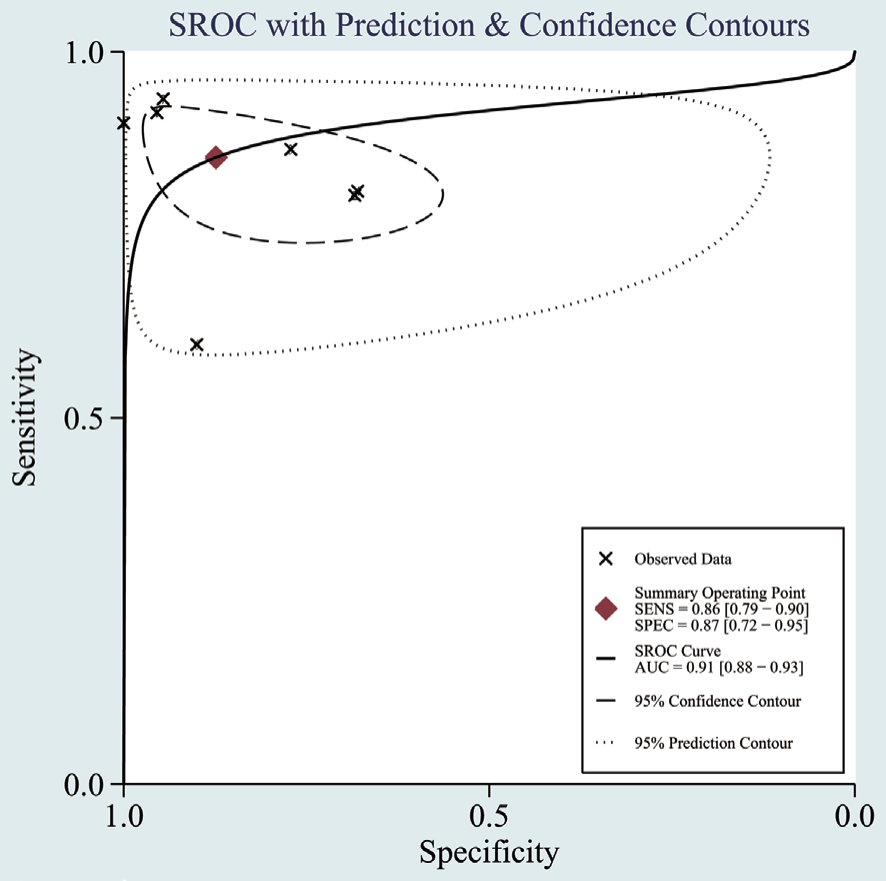
**Summary receiver operating characteristic (SROC) curve**. Observed data obtained from meta-analysis (Cheng et al., 2014; Kumar et al., 2014; Lau et al., 2014; Leidinger et al., 2013; Muller et al., 2014; Tan et al., 2014a,b).

**Figure 4:**
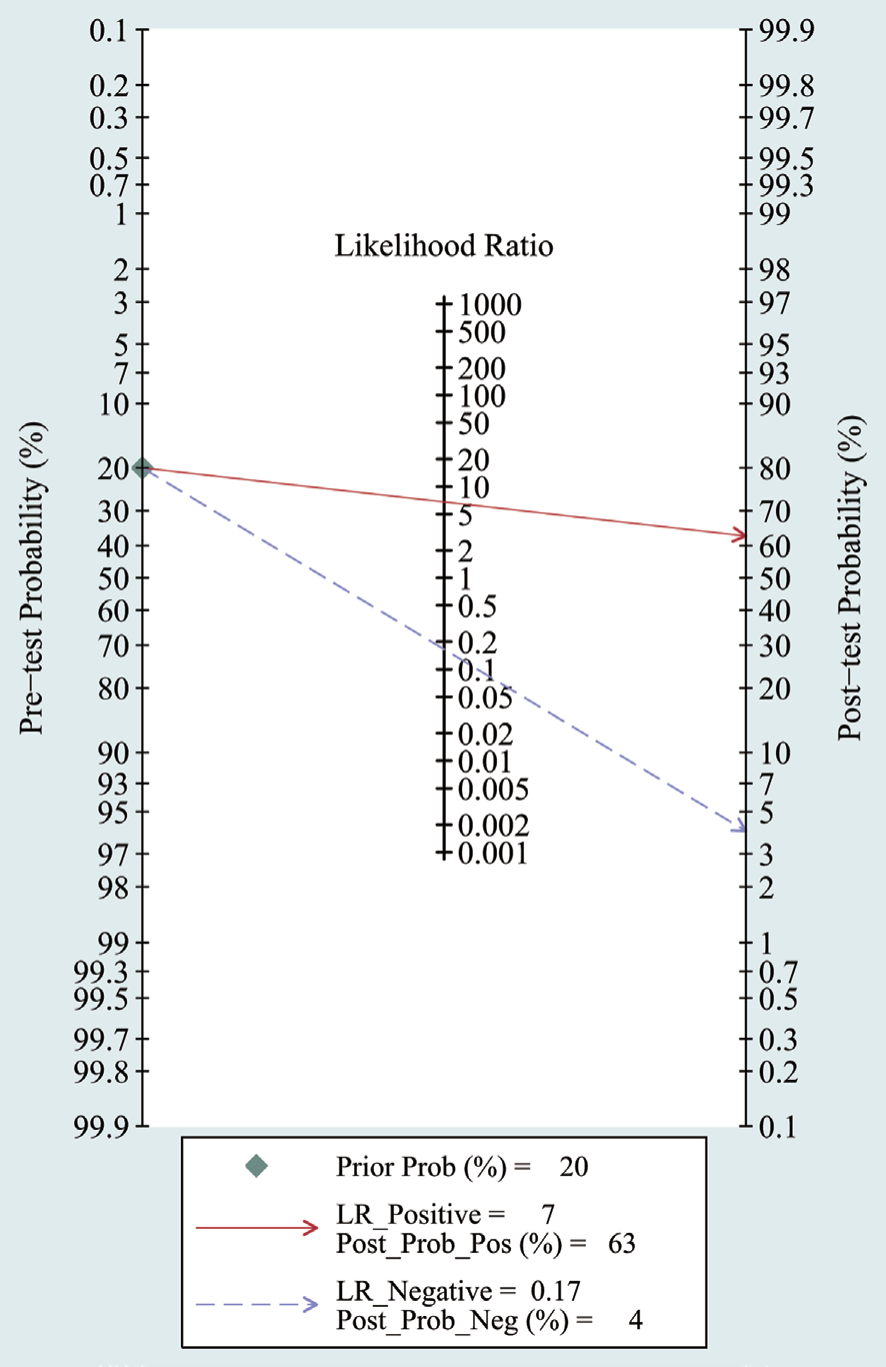
**Fagan’s Nomogram for assessment of post-test probabilities**. Observed data obtained from meta-analysis (Cheng et al., 2014; Kumar et al., 2014; Lau et al., 2014; Leidinger et al., 2013; Muller et al., 2014; Tan et al., 2014a,b).

The heterogeneity *I*^2^ value for inter-study variability is 78.39%. As a result, the summary assessment of miRNAs in the diagnosis of AD showed that the pooled sensitivity was 0.86 (95%CI: 0.79–0.90) and the pooled specificity was 0.87 (95%CI: 0.7–0.95) (Table 4; Figure 2). The area under the summary receiver operating characteristic curve was 0.91 (Figure 3). The PLR was 7 and the NLR is 0.17, as demonstrated in the Fagan’s nomogram in Figure 4. Moreover, the combined diagnostic odds ratio was 28.29.

**Table 4:** **Accuracy estimates of included studies**.

Study	Reference	Sensitivity (95%CI)	Specificity (95%CI)	PLR (95%CI)	NLR (95%CI)	DOR (95%CI)
1	Leidinger et al. (2013)	0.92 (0.80, 0.98)	0.95 (0.77, 1.00)	20.17 (2.96, 137.12)	0.087 (0.034, 0.224)	231.00 (24.29, 2196.30)
2	Tan et al. (2014a)	0.80 (0.73, 0.86)	0.68 (0.60, 0.76)	2.54 (1.99, 3.25)	0.287 (0.206, 0.400)	8.86 (5.28, 14.88)
3	Cheng et al. (2014)	0.87 (0.60, 0.98)	0.77 (0.60, 0.90)	3.79 (1.99, 7.19)	0.173 (0.047, 0.636)	21.94 (4.07, 118.28)
4	Tan et al. ([Bibr B47][Bibr B46])	0.81 (0.72, 0.88)	0.68 (0.60, 0.75)	2.53 (1.97, 3.25)	0.280 (0.186, 0.422)	9.03 (4.98, 16.39)
5	Lau et al. (2013)	0.90 (0.77, 0.97)	1.00 (0.85, 1.00)	42.86 (2.75, 666.92)	0.109 (0.046, 0.262)	391.67 (20.16, 7610.80)
6	Muller et al. (2014)	0.60 (0.36, 0.81)	0.90 (0.68, 0.99)	6.00 (1.54, 23.44)	0.444 (0.255, 0.775)	13.50 (2.43, 74.87)
7	Kumar et al. (2013)	0.94 (0.79, 0.90)	0.95 (0.82, 0.99)	17.31 (4.48, 66.83)	0.068 (0.018, 0.261)	253.75 (33.63, 1914.40)
Total		0.86 (0.79, 0.90)	0.87 (0.72, 0.95)	4.29 (2.62, 7.03)	0.206 (0.133, 0.319)	28.29 (11.095, 72.16)

Furthermore, in order to decrease the mixing affects in interpreting the results, we performed another analysis using body fluid miRNAs, including plasma, serum, and CSF miRNAs in the diagnostic test of AD. This analysis shows similar results compared to the former comprehensive meta-analysis (Figures S2 and S3 in Supplementary Material).

### Publication Bias

**Figure 5:**
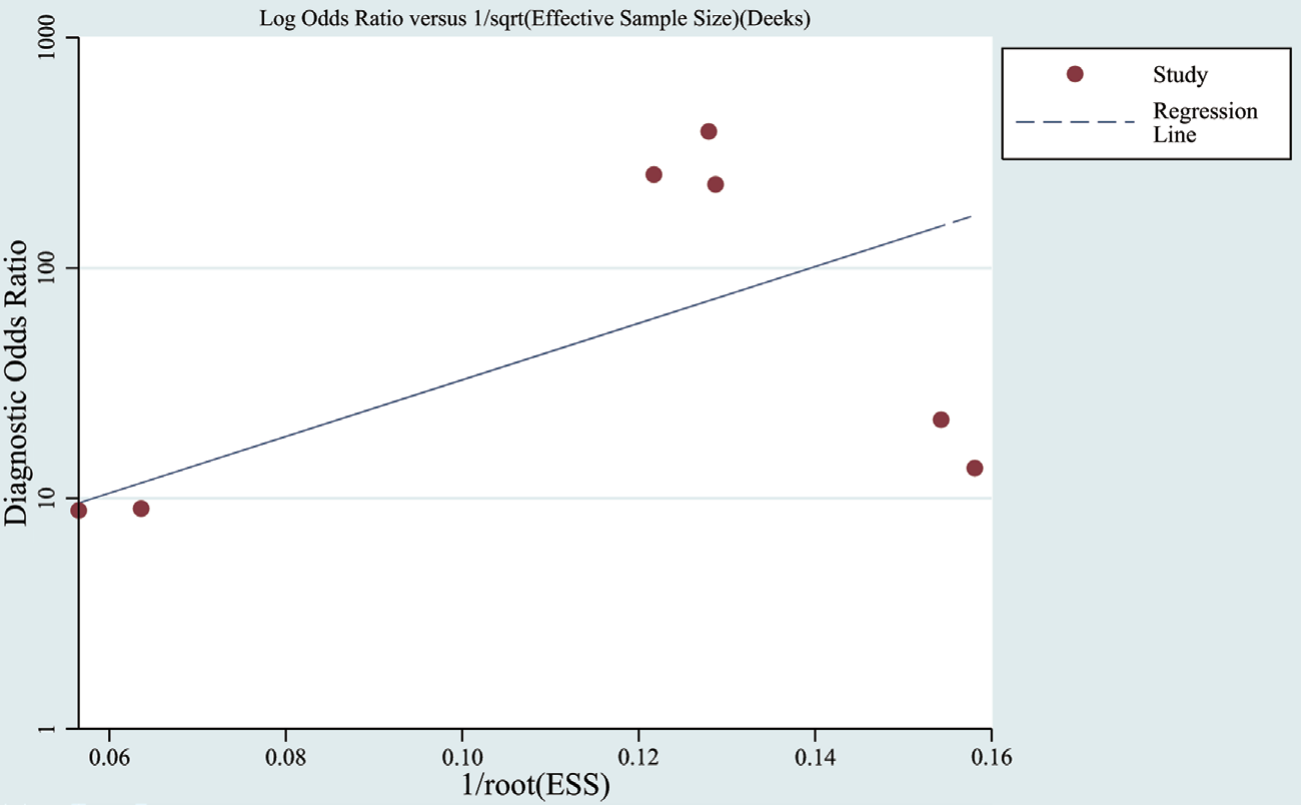
**Funnel graph for the assessment of publication bias**. Observed studies obtained from meta-analysis (Cheng et al., 2014; Kumar et al., 2014; Lau et al., 2014; Leidinger et al., 2013; Muller et al., 2014; Tan et al., 2014a,b).

Funnel plots were used to assess the publication bias of the included studies in meta-analysis as listed in Figure 5. Furthermore, in a quantitative method, the Deek’s test was performed and the *p* was 0.07.

## Discussion

The development of sensitive and specific biomarkers for diagnosing AD remains a great challenge, especially with most patients being asymptomatic at the early stage (Iacono et al., 2008). Given that miRNAs plays a critical role in neural development and neurological diseases, it is reasonable to assume that they may serve as putative markers of AD. The overall aim of this investigation was to evaluate the diagnostic value of miRNAs in AD and examine the correlation between AD associated neurodegeneration and the expression of miRNAs in CSF, hippocampus or serum, especially in the early subclinical stages of AD.

To date, the correlation of miRNA dysregulation with AD has been widely studied. However, the results of these studies had not always been consistent and no consensus has yet been reached. We are undoubtedly just at the beginning of exploring this field, and there remains an immense amount of work to fully define the relationship between miRNAs and AD. First, we have reviewed more than 50 miRNAs, which expressed aberrantly in biofluid and brain of AD. Most of miRNAs are downregulated; by contrast, some specific miRNAs are significantly upregulated, such as miR-29, miR-339, and miR-214. Then, we further explored the dysregulation of miRNAs in the pathological process of AD including APP metabolism, Tau pathology, neuroinflammation, and apoptosis. Many studies suggest that miRNAs serve as critical regulators in those signaling pathways and the dysregulated miRNAs could influence gene transcription and protein expression and induce the spread of neurodegeneration (Faghihi et al., 2010; Schonrock and Gotz, 2012).

In meta-analysis, the quality of most studies was relatively good to a certain extent with QUADAS scores ranged from 10 to 13 and the pooled sensitivity was 0.86 and specificity was 0.87 with an AUC of 0.91. We also found that the DOR upon combining the pooled sensitivity and specificity was 28.29, which further supports miRNAs as a potential diagnostic tool for AD. For assessment of post-test probabilites, Fangan’s Nomogram was used, and its results indicated that the PLR was 7, suggesting a patient with AD is seven times more likely to have positive results than a normal person, which reveals that both likelihood ratios and post-test probabilities were moderate. In publication bias assessment, according to our selection criteria in meta-analysis, only clinical studies were included and some data such as reports, conference abstracts, and non-English and non-Chinese literature were excluded, which probably causes publication bias to some degree, but there is no significant evidence for publication bias in the Deek’s test.

However, several limitations complicate the interpretation of our meta-analysis. First, heterogeneity is a major problem in interpreting the results. miRNAs expression may vary between CSF, hippocampus, and peripheral blood, which may result in different diagnostic efficiency of miRNAs for AD. Additionally, the studies utilized different methods for quantitative detection and normalization in miRNA analysis, which may account for the differences reported across different studies. Finally, the relatively small sample size of each study as well as the limited number of eligible studies would also affect the statistical power of our results.

In conclusion, the present meta-analysis suggests a promising role of miRNAs in the detection of AD. Compared to other existing markers, the miRNA biomarkers has the advantage of being easily accessible and rapidly analyzed and could provide insight to the early diagnosis of AD, and recent studies are demonstrating improving sensitivity and specificity. However, currently, there still lacks a clear classification for tissue based or blood-based miRNAs. Future prospective, multi-centered, large-scale clinical trials will further aid the establishment of miRNA combinations as diagnostic biomarkers for AD. Moreover, further studies on to further elucidate the pathophysiological interaction between miRNAs and AD-related proteins are also required.

## Author Contributions

Y-BH did the literature research, data acquisition, statistical analysis, and wrote the manuscript. C-BL and NS provided insightful thoughts to study concepts and wrote the manuscript. YZ assisted with the literature review and data collection. R-JR, GW, and S-DC designed the study, revised the manuscript, and approved the final version.

## Conflict of Interest Statement

The authors declare that the research was conducted in the absence of any commercial or financial relationships that could be construed as a potential conflict of interest.
