# Corrigendum: Diagnostic Value of microRNA for Alzheimer's Disease: A Systematic Review and Meta-Analysis

**DOI:** 10.3389/fnagi.2017.00035

**Published:** 2017-02-23

**Authors:** Yong-Bo Hu, Chun-Bo Li, Ning Song, Yang Zou, Sheng-Di Chen, Ru-Jing Ren, Gang Wang

**Affiliations:** ^1^Department of Neurology, Neuroscience Institute, Ruijin Hospital, Shanghai Jiao Tong University School of MedicineShanghai, China; ^2^Shanghai Key Laboratory of Psychotic Disorders, Shanghai Mental Health Center, Shanghai Jiao Tong University School of MedicineShanghai, China; ^3^St. George HospitalSydney, NSW, Australia

**Keywords:** Alzheimer's disease, miRNA, diagnostic value, systematic review, meta-analysis

In the originally published article, due to the author's misunderstanding of methods of RNA isolation, one of included studies (Leidinger et al. 2013) in Table 3, the source of miRNAs ie. (specimen column) should be “whole blood cell” instead of “plasma”. And in Table 1, the citation (Leidinger et al. 2013) should be removed. The revised tables has been provided below. This error does not change the scientific conclusions of the article. The authors regret the error.

**Table 1:** **Systematic review: miRNA dysregulation in different parts**.

**Brain-based miRNA**		**CSF-based miRNA**	**Blood-based miRNA**		
**Cortex**	**Hippocampus**		**Plasma**	**Serum**	**PBMC**
miR-129-5p miR-27a-3p	miR-132-3p miR-128	miR-34a, miR-125b miR-146a, miR-29a	miR-34a/c miR-146a	miR-137 miR-18c	miR-34a miR-181b
miR-92b-3p	miR-136-5p	miR-27a-3p,	miR-128	miR-9	miR-200a
miR-200a miR-148	miR-138-5p miR-145	miR-24, miR-126, miR-10a/b, miR-16	miR-132 miR-29a/b	miR-29a let-7f	let-7f
miR-370 miR-409-5p miR-127-5p miR-496 miR-633 miR-874	miR-124-3p miR-129-5p miR-129-2-3p miR-487 miR-370 miR-409-5p miR-487	miR-138, miR-141 miR-143, miR-151 miR-181a/c miR-191, miR-194 miR-195, miR-204 miR-205, miR-214 miR-221, miR-338	miR-874 miR-134 miR-323-3p miR-382 miR137 miR181c	miR-29b miR-126 miR-34a miR-181b	
Lau et al. (2013), Delay et al. (2012), Bekris et al. (2013)	Lau et al. (2013), Delay et al. (2012)	Bekris et al. (2013), Cogswell et al. (2008), Kiko et al. ( 2014), Muller et al. (2014), Sala Frigerio et al. (2013), Burgos et al. (2014)	Kumar et al. (2013), Bekris et al. (2013), Bhatnagar et al. (2014), Kiko et al. (2014)	Cheng et al. (2014), Tan et al. (2014a,b), Geekiyanage et al. (2012)	Schipper et al. (2007)

**Table 3:** **Summary of included studies**.

**Study**	**Author**	**No. of patients**	**No. of controls**	**Specimen**	**TP**	**FP**	**FN**	**TN**	**QUADAS**	**miRNA profile**
1	Leidinger et al., 2013	48	22	Whole blood cell	44	1	4	21	10	miR-112, 161,5010-3p,26a-5p, 1285-5p, 151-3p
2	Tan et al., 2014a	158	155	Serum	127	49	31	106	13	miR-98-5p,885-5p,483-3p,191-5p,let-7d-5p
3	Cheng et al., 2014	15	35	Serum	13	8	2	27	10	miR-1306-5p,342-3p,15b-3p
4	Tan et al., 2014b	105	150	Serum	85	48	20	102	12	miRNA-125b
5	Lau et al., 2013	41	23	Hippocampus	37	0	4	23	13	miR-132-3p, 128, 136-5p,138-5p,124-3p,129-5p
6	Muller et al., 2014	20	30	CSF	12	2	8	18	13	miR-16
7	Kumar et al., 2013	31	37	Plasma	29	2	2	35	11	miR-545-3p,let-7g-5p
**Total**		418	442					

## Conflict of interest statement

The authors declare that the research was conducted in the absence of any commercial or financial relationships that could be construed as a potential conflict of interest.

