# Supplementary material for: Diagnostic Value of microRNA for Alzheimer’s Disease: A Systematic Review and Meta-Analysis
Source: Front Aging Neurosci. 2016 Feb 9;8:13. doi: 10.3389/fnagi.2016.00013 (PMC4746262; doi:10.3389/fnagi.2016.00013)
Supplement: Supplementary file 1 [file data_sheet_1.docx]

Figure S1：dysregulated miRNAs across the Braak stages

(n=41, compared to the control)( Lau et al., 2013).


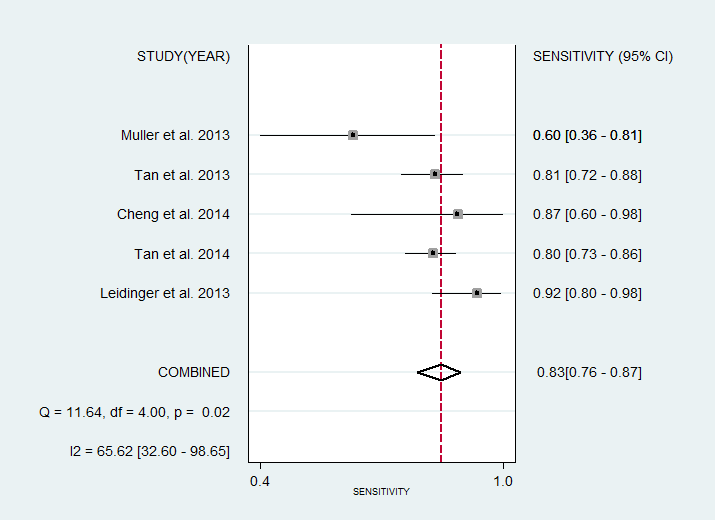

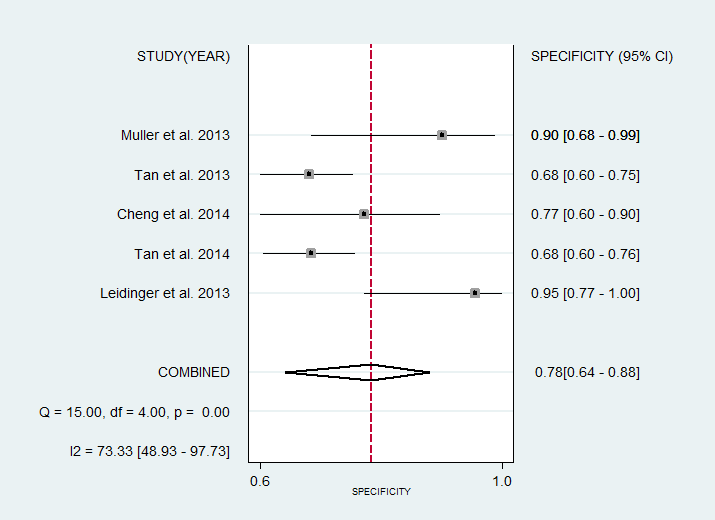


Figure S2: Forrest plot of estimates of sensitivity and specificity (body fluid).


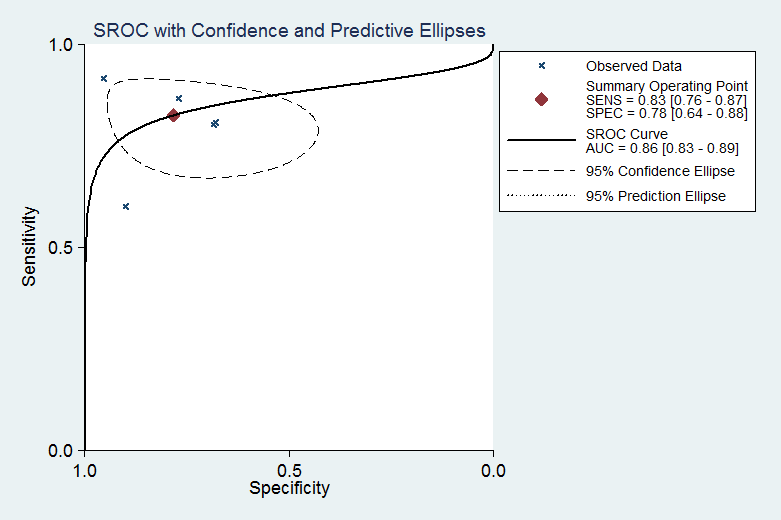


Figure S3**.** Summary receiver operating characteristic (SROC) curve (body fluid). Observed data obtained from meta-analysis (Leidinger et al., 2013;Tan et al., 2014a; Cheng et al., 2014; Tan et al., 2014b; Muller et al., 2014;).
